# Assessment of Donkey (*Equus asinus africanus)* Whole Blood Stored in CPDA-1 and CPD/SAG-M Blood Bags

**DOI:** 10.3390/biology10020133

**Published:** 2021-02-08

**Authors:** Isabella Oliveira Barros, Rejane Santos Sousa, Marcondes Dias Tavares, Renato Otaviano Rêgo, Paulo Ricardo Firmino, Francisco Jocelho Alexandre Souza, Maria Rociene Abrantes, Antonio Humberto Hamad Minervino, Carolina Akiko Sato Cabral Araújo, Enrico Lippi Ortolani, Raimundo Alves Barrêto Júnior

**Affiliations:** 1 Department of Veterinary Science, Federal University of Paraíba, Rodovia PB 079, Km 12, Areia 58397-000, Brazil; doutorabella@hotmail.com; 2 Institute of Studies of the Humid Tropic, Federal University of the South and Southeast of Pará, Rua Alberto Santos Dumont, s/n, Xinguara 68557-335, Brazil; rejane.santossousa@gmail.com; 3 Department of Animal Science, Federal Rural University of the Semi-Arid, Av. Francisco Mota, Mossoró 59625-900, Brazil; marcondesmv@hotmail.com (M.D.T.); renato_otaviano@yahoo.com.br (R.O.R.); pauloricardo83@hotmail.com (P.R.F.); fjocelho@outlook.com (F.J.A.S.); rocienevet3@hotmail.com (M.R.A.); 4 Laboratory of Animal Health, LARSANA, Federal University of Western Pará, UFOPA, Rua Vera Paz No Number, Santarém 68040-080, Brazil; 5 Department of Veterinary Medicine, Federal Rural University of Pernambuco, Rua Dom Manuel Medeiros, Recife 52171-900, Brazil; mvcarolcabral@gmail.com; 6 School of Veterinary Medicine and Animal Science, University of São Paulo, Av. Dr. Orlando Paiva, 97, São Paulo 05508-270, Brazil; ortolani@usp.br

**Keywords:** red blood cells, transfusion, equids, stored blood

## Abstract

**Simple Summary:**

The development of conservative solutions was essential to store blood for different periods, but there is no blood bag available for animals. Currently, the solution containing citrate, phosphate dextrose, and adenine (CPDA-1) and the solution containing citrate, phosphate, and dextrose plus mannitol and sodium chloride (CPD/SAG-M) are the most used in human blood conservation. In this study, we propose to evaluate whether the CPDA-1 and CPD/SAG-M blood bags designed for humans are efficient for the conservation of donkey whole blood for 42 days. During storage, both blood bags resulted in mild alterations in the stored blood, but the two bags were efficient and very similar in preserving donkey blood for up to 42 days. Both types of human-designed blood bags can be used for donkey transfusion medicine.

**Abstract:**

Hemotherapy using whole blood and its components is being increasingly used in veterinary therapy. Since it is important to store animal blood while maintaining acceptable hematological, blood gas, and biochemical characteristics, increasing our knowledge of available technologies for strategic blood storage is imperative. Thus, we aimed to assess the hematological, blood gas, and biochemical changes in donkey whole blood using blood bags with two different types of storage agents. Eight adult healthy male donkeys were used; 900 mL of blood was collected from each, with 450 mL stored in citrate-phosphate-dextrose and adenine bags (CPDA-1) and 450 mL stored in bags containing citrate-phosphate-dextrose, adenine, mannitol, and sodium chloride (CPD/SAG-M). Both bags were kept refrigerated between 1 and 6 °C for 42 days. Blood samples were removed from the bags eight times (T): T0 (immediately after blood collection), T1, T3, T7, T14, T21, T35, and T42 (1, 3, 7, 14, 21, 35 and 42 days after storage). Hematological, blood gas, biochemical, and microbiological parameters were assessed. The CPDA-1 bags had a higher packed cell volume when compared to CPD/ SAG-M. The red blood cell count reduced by around 19% in both the bags due to hemolysis, which was confirmed by an increase in plasma hemoglobin. The white blood cell count; pH; concentrations of glucose, sodium, bicarbonate, and 2,3 diphosphoglycerate were reduced in both bags. Meanwhile, pO_2_, pCO_2_, lactate dehydrogenase, and levels of potassium increased in the CPDA-1 and CPD/SAG-M bags. Blood bags were efficient for the storage of donkey blood for up to 42 days.

## 1. Introduction

Whole blood transfusion plays an important role in clinical veterinary procedures and during surgery in large animals. It is known that blood transfusion is a type of therapy often used in cases of serious anemia, for the restoration of blood volumes and coagulation components, the re-establishment of the ability of oxygen transport in tissues, and plasma protein supplementation [1,2,3,4]. In horses, transfusion is widely used in some emergency cases [5]. However, studies about blood transfusion in large animals, especially those related to preservation methods for blood, are scarce. To our knowledge, there is no study that evaluates donkey blood conservation. Hence, studies aiming to improve blood storage conditions will contribute to our understanding of blood transfusion. The preservation time could thus be extended without compromising the maintenance of the hematological and biochemical parameters [6].

The blood storage bags used in veterinary medicine were developed for the storage of human blood, however studies have been evaluating the viability of such blood bags for the storage of blood of different animal species [7,8]. The CPDA-1 bags contain a solution of citrate, phosphate, dextrose, and adenine (63 mL) that allows the storage of human blood for 35 days, while the CPD/SAG-M bags, in addition to citrate, phosphate, and dextrose (63 mL) have an additive solution in a satellite bag containing saline, adenine, and mannitol (100 mL) that provides a greater supply of energy and phosphate for the survival of red blood cells. CPDA-1 bags are cheaper and easier to find than CPD/SAG-M [9]. 

Blood bags containing citrate-phosphate-dextrose and adenine (CPDA-1) are the most commonly used ones to preserve blood under refrigeration in veterinary medicine [10]. CPDA-1-type blood bags have better red blood cell preservation properties than ACD (acid citrate dextrose), allowing whole blood storage for canines, [11] equines, [5,12] bovines, [13] sheep, [10] goats, [9] and buffaloes [14] for up to 35 days. In small animal transfusion medicine, a reduction in storage time has been observed, with the maximum storage time varying between 21 and 28 days [15,16,17]. The objective of this work was to quantify and assess the effect of the preservation process on donkey whole blood storage in CPDA-1 and CPD/SAG-M (citrate-phosphate-dextrose, adenine, mannitol, and sodium chloride) bags by assessing the hematological, blood gas, and biochemical parameters over 42 days of storage. Our hypothesis is that bags containing CPDA-1 and CPD-SAG/M, developed and used for human medicine, can preserve donkey blood for 42 days of storage. 

## 2. Materials and Methods

The animals used in this study were handled with the best practices of animal welfare and this work was approved by the Animal Ethic commission from University Federal do Semiarido, UFERSA. The approval protocol number is 3091.000795/2011-41.

We performed a prospective clinical trial with paired blood samples placed in two blood bags with distinct types of preservatives: CPDA-1: 63 mL solution of citrate, phosphate, dextrose (CPD), and adenine; CPD/SAG-M: 63 mL of CPD in the primary bag and 100 ml solution of mannitol and sodium chloride in the satellite bagEight healthy, adult male, non-castrated Northeast breed donkeys were used. They had a mean weight of 120 ± 20 kg and were 1 to 3 years of age. Their health was verified by a physical examination and hemogram. The animals were kept in a collective stall, where they were fed a diet composed of fresh grass (*Panicum maximum*) that was cut daily and offered to animals according to 3.0% of their body weight (on dry matter basis). Mineral mixture and water were supplied ad libitum. All the animals were worm-free and underwent a period of 20 days of adaptation. Subsequently, the collection and storage of blood was conducted with both CPDA-1 and CPD/SAG-M blood bags. 

In order to collect blood, the animals were manually restrained and 900 mL of whole blood was collected from each animal, with 450 mL stored in a sterile bag containing CPDA-1 (eight experimental units) and 450 mL in a sterile bag with CPD/SAG-M (eight experimental units). Minutes before blood collection from the CPD/SAG-M bags, the additive solution present in the satellite bag was transferred to the main bag and the whole blood collection was conducted. After collection, the blood was placed in a refrigerator at a temperature between 1 and 6 °C for 42 days. Blood was placed into bags alternately (i.e., changing the bag type order in each donkey). After blood withdrawal, the tube that connects the sampling needle to the blood bag was sealed with a sealer. At each collection, the bags were homogenized, and this tube was opened to draw blood. 

The laboratory assessment of the whole blood collected and stored was conducted at nine different periods: T0 (immediately after collection), T1, T3, T7, T14, T21, T28, T35, and T42 days after collection. Before the assessment of the stored blood at the mentioned days, the bags of blood were homogenized manually and 7 mL of blood from the interior of the bags was removed for the measurement of hematological (2 mL), blood gas (1 mL), and biochemical variables (3 mL) and microbiological culture (1 mL). For each blood sampling, sterile tubes and syringes were used and the bag was handled with sterile protocols to avoid any type of contamination. Blood gas samples were obtained anaerobically to avoid interference with the results.

The assessment of red blood cell and leucocyte counts was determined manually in a Neubauer chamber by macro-dilution. The packed cell volume was obtained using a centrifuge for microhematocrit (12,000× *g* for five minutes). The concentration of plasma hemoglobin was determined through samples of plasma using the cyanmethemoglobin method.

The level of hemolysis was assessed through the hemoglobin concentration present in the whole blood, the plasma hemoglobin concentration, and the packed cell volume (PCV). The determination of hemolysis level was conducted according to the protocol in the study by Wardrop et al. [11].

The measurements of pH, partial pressure of O_2_ (PO_2_), partial pressure of CO_2_ (PCO_2_), bicarbonate (HCO_3_^−^), sodium, and potassium were conducted with 2 mL of blood, through a blood gas portable apparatus (i-Stat, Abbott Laboratories, Chicago, IL, USA) using commercial cartridges. This equipment had shown a good agreement with the standard analyzer for the venous blood of donkeys [18]. The glucose and lactate concentrations and lactate dehydrogenase (LDH) activity were determined in an automatic biochemistry analyzer (Rx Daytona, Randox, Antrim, UK). The concentration of 2,3 diphosphoglycerate (2.3 DPG) was determined using commercial kits (Roche Diagnostics, Basel, Switzerland) for ultraviolet light spectrophotometry. Analytical procedures were validated using commercial quality controls test serum. For microbiological evaluation to identify possible contamination of the blood bags that would interfere with the results, at each time point blood samples from the bags were submitted to aerobic bacteriological culture at 36 °C in blood agar culture medium with the evaluation of bacterial growth in 48 h. 

Statistical analysis was performed with SAS statistics software. The Kolmogorov–Smirnov test for normality was performed, followed by the PROC ANOVA for comparison among bags (CPDA-1 × CPD/SAG-M) and Bonferroni’s test for comparison between T0 (baseline) and other times. Pearson’s correlation was used to assess the relationship between two variables. A minimum level of 5% significance was adopted in this study. 

## 3. Results

The PCV values were higher in the CPDA-1 bags than the SAG-M bags at all times (Table 1). In both the bags, the PCV increased at T42 when compared to T0. There was a difference in the number of red blood cells between both bags; the amount of red blood cells in the SAG-M bag was lower than that in the CPDA-1 at all points of assessment. In the SAG-M bags, the decrease in erythrocytes occurred at T35; however, in CPDA-1 the decrease occurred at T42.

There was no difference in the plasma hemoglobin concentration between the bags. However, there was an increase in plasma hemoglobin in the CPDA-1 and SAG-M bags at T35 and T21, respectively, when compared to baseline (T0). The percentage of hemolysis did not differ between the two bags but increased from baseline at T21 in the SAG-M bag and at T35 in the CPDA-1 bag. An increased leucocyte count was observed in the CPDA-1 bag until T21 compared to the SAG-M bag. Compared to T0, a reduction in the leucocyte count was observed from T14 and onwards in both bags. 

There was no difference in blood pH between the bags, but over time a pH reduction (*p* < 0.05) started at T7 in SAG-M and at T14 in CPDA-1 when compared with baseline (Table 2). Between bags, higher values of carbon dioxide pressure at time points T3, T7, T14, T21, and T28 were observed. In these periods, the values of pCO_2_ in the CPDA-1 bags were higher than those in the SAG-M bags. The values of pCO_2_ increased in the CPDA-1 and SAG-M bags at T7. A high negative correlation between pCO_2_ and pH, with r = −0.92 for CPDA-1 and r = −0.86 for SAG-M, was observed.

Higher values of oxygen pressure at the times T3, T7, T21, and T28, were observed for CPDA-1 than for SAG-M. There was an increase in pO_2_ at T7 compared to T0 in the CPDA-1 bag, but in the SAG-M bag this only occurred at T28. The values of bicarbonate in the CPDA-1 bags were higher than those in the SAG-M bags at all periods of storage. After T42 and T35, the levels of bicarbonate decreased in the SAG-M and CPDA-1 bags.

The concentration of 2,3 DPG did not differ between the blood stored in the CPDA-1 and SAG-M bags; however, over time it was observed that both bags had smaller values of 2,3 DPG at T42 compared to T0 (Table 3). The glucose concentrations at T0, T1, T3, T7, T14, and T21 were higher in CPDA-1 compared to SAG-M. From T28 onwards, the glucose concentration did not differ between the bags. In CPDA-1 and SAG-M, a decrease (*p* < 0.05) in glucose concentration (compared to baseline) occurred at T21.

The blood stored in the CPDA-1 bags had higher concentrations of lactate than that in SAG-M until T21. By the end of the study, the concentration of lactate did not differ between the bags. Higher values of lactate dehydrogenase (LDH) were found from T1 to T21 for blood conditioned in the CPDA-1 bags compared to blood stored in the SAG-M bags. Higher values of LDH were detected at T42 for both bags. The concentration of sodium was higher at almost all times (except T7) in the SAG-M bag than in the CPDA-1 bag. When each bag was assessed over time, it was observed that the sodium concentrations in CPDA-1 were reduced from baseline values at T21 and onwards and in SAG-M at T14 and onwards. The potassium concentration between bags increased from T14 until T42, and the concentrations in the CPDA-1 bags were higher than those in the SAG-M bags (*p* < 0.05). The potassium concentration in the CPDA-1 and SAG-M bags increased at T1. The relation between pH and levels of potassium in the CPDA-1 and SAG-M bags showed a high negative correlation: r = −0.89 for the CPDA-1 bags and r = −0.83 for the SAG-M bags. 

Over 42 days of storage, we did not observe any bacterial growth in the anaerobic medium from blood samples obtained from both the CPDA-1 and SAG-M bags.

## 4. Discussion

The number of red blood cells was higher in the CPDA-1 bags from T0 compared to in the SAG-M bags. The higher volume of preservation solution in the SAG-M bag (163 mL) compared to in the CPDA-1 bag (63 mL) justifies this difference. This effect was also observed in studies on other species [19]. The drop in the number of red blood cells over the analysis time occurred due to red blood cell lysis, which may have been due to storage lesions or simply due to the natural death of red cells, since the sampled whole blood had cells with different ranges of age with a reduced lifespan (145–155 days for horse) [20].

Similar to what happened with the number of red blood cells, the PCV was lower for the SAG-M bag due to the higher solution volume. However, an increase in the PCV was observed over time in both bags, which occurred due to swelling red blood cell as a result of storage in the preservation solution. Although it appears odd, this PCV increase can be explained by observing the mean corpuscular volume (MCV), which increased at T42 by 26 and 38% in CPDA-1 and SAG-M, respectively. Since blood cells are swollen, the red cells total volume can increase, which reflects in the PCV measured using the microhematocrit method. In horses, this parameter behaved the same way in both types of blood bags [5,12]. 

There was a significant difference in MCV between both the bags on the last day of assessment, while the individual analysis showed an increase in MCV at T42. The cell increase could have occurred mainly due to failures in the sodium and potassium pumps, which in turn allowed the exit of intracellular potassium and the entrance of sodium [21] promoting an increase in the hypertonicity of the intracellular medium and consequently the entry of water into the cell [22]. The MCV value of horse blood estimated by Mudge et al. [12] in CPDA-1 bags and by Niinisto et al. [5] in SAG-M bags were lower than those of donkey blood in a comparison of the same storage times. 

There was an increase in the plasma hemoglobin concentration in each bag over time. Over the preservation period, storage lesions occurred progressively, and hemoglobin was released as the erythrocytes broke apart. This was associated with the detachment of vesicles from the surface of the erythrocytes with hemoglobin in its interior, which also contributed to an increase in the plasma concentration of this pigment [6]. The increase in plasma Hb is accompanied by an increase in extracellular potassium and LDH, which, in this case, was higher in CPDA-1 than in SAG-M bags and was related to the level of red blood cell hemolysis in the interior of the bags, which tends to increase over time. In horse blood stored with CPDA-1 and SAG-M, an increase in the concentration of hemoglobin after 30 days of storage was observed [5,12]. The concentration of plasma hemoglobin acceptable for humans after 35 days of storage is 0.8 g/dL. In our study, the plasma hemoglobin remained low until the end of the experiment in both the bags.

The reduction in the leucocyte count occurred in the stored blood in both bags. Biochemical changes in blood during storage could cause a disequilibrium on the membranes of the cytoplasmic organelles of leucocytes, with the consequent rupture and release of several enzymes capable of destroying them. Over time, these cytoplasmic changes may intensify and cause the number of leucocytes to decrease. In addition, it is believed that leucocyte reduction improves the energetic use of red blood cells, which will not need to compete with leucocytes for energy [23].

The degree of hemolysis of red cells increased for both blood bags over the time of storage. Niinisto et al. [5] obtained a mean level of hemolysis of 0.27% after 35 days of storage of equine red blood cells in SAG-M bags. This result was marginally higher than what was found in this study (0.25%) when comparing the same times of storage. Although the SAG/M blood bag is designed for keeping P low, high intracellular pH, and high ATP, thus reducing hemolysis and therefore extending the storage time, we did not found differences between the blood bags (*p* > 0.05). The SAG/M blood bag was developed for human blood. In our study, as in other studies with animal species, this bag did not result in an additional improvement of stored blood quality [9]. This may be related to the specificity of animal blood, and animal-designed blood bags may be required to improve blood parameters. Another possibility is that SAG/M is used to store red blood cell concentrate and not whole blood in human medicine. The reduction in the number of leukocytes and platelets that occurs with the removal of plasma and the addition of the additive solution (SAG-M) provides more energy, higher stability of the membrane, and higher intracellular pH of the red blood cells.

Despite the observation of an increase in the degree of hemolysis, the values were kept within the limit proposed for the storage of human blood, which is 0.8% in Europe and 1% in the United States and Brazil. Since this is the first study concerning the storage of erythrocytes, the authors considered the hemolysis rate to be a fundamental marker of blood preservation [22,24].

The changes in the acid-basic state in whole blood is described in horses, since the pH of preserving solutions is acidic [12]. The results showed a gradual pH reduction over the assessment period for both bags. The pH reduction occurred at different times for each bag; in the CPDA-1 bag this occurred from T35, while in the SAG-M bag this reduction was seen at T42. The metabolic reactions of erythrocytes in the interior of bags tend to change the blood pH to acidic. This is due to anaerobic metabolism, which produces lactate corresponding to around 95% of total red blood cell metabolism, resulting in acid production [13]. The blood pH reduction coincided with the increase in the concentration of blood lactate in both bags, as confirmed in other studies [10,13].

Considering the same period of assessment (28 days), our pH data (6.84 CPDA-1; 6.83 SAG-M) are among the values suggested by Mudge et al. [12] and Niinisto et al. [5] when using CPDA-1 and SAG-M bags for the preservation of horse blood, respectively. If the pH values are compared at the same time period (35 days) in CPDA-1 bags, for example, it is possible to note that the donkey blood kept the pH (6.83) level more elevated than some ruminants such as cattle (6.53) and sheep (6.72) [10]. According to Hess and Greenwalt [6], a pH below 6.2 represents the minimum limit that the cell continues to metabolize in stored blood. The lowest pH values observed in this study were 6.78 (CPDA-1) and 6.71 (SAG-M), showing the viability of donkey blood after 42 days of preservation concerning this variable.

The decrease in blood pH stimulated degradation and reduced the production of 2,3 DPG in both bags. The reduction in 2,3 DPG promotes an increase in the affinity of hemoglobin for oxygen (Bohr effect) [25,26]. 

The increase in the levels of pCO_2_ until T35 (CPDA-1) and T21 in SAG-M was probably due to the neutralization of lactate produced by the red blood cell metabolism, which is combined with bicarbonate, resulting in the final products of water and CO_2_, thus increasing the pCO_2_. CO_2_ may be linked to hemoglobin and cellular exhaustion, in which the production velocity of this gas was reduced, associated with a loss of carbon dioxide amount that crosses the plastic in the bag, as mentioned by Hess and Greenwalt [6]. Hence, the stabilization of the levels of this gas at T35 in CPDA-1 and T21 in SAG-M may be justified.

Between bags, higher values of pO_2_ were observed in the CPDA-1 bags at times T3, T7, T21, and T28 (*p* < 0.05). At all these times, there was an increase in pO_2_ in CPDA-1 concerning SAG-M. In the CPDA-1 bags, the increase in the partial pressure of oxygen was observed only at T7, while in SAG-M it was observed at T14. This increase in pO_2_ in donkey blood was also observed in cattle [13] and sheep blood, both stored in CPDA-1 [10]. The increase in pO_2_ is probably due to the reduction in 2.3 DPG, which led to an increase in the affinity of hemoglobin for oxygen.

The plasma bicarbonate values were different between bags over all periods of storage, and were always reduced in SAG-M. Bicarbonate is a weak base and is responsible for more than 50% of the buffer capacity of the extracellular medium [27]. This suggests that this reduction in the blood values of HCO_3_ may be due to the consumption of this ion in the control of acidity, arising from an increase in lactate.

The glucose levels in the SAG-M bag were kept lower than those of the CPDA-1 bag until 28 days of storage: after this period, there was no difference among glucose levels. Despite the glucose values remaining smaller in the SAG-M bags than in the CPDA-1 bags, when we compared the final period of storage these values were less reduced in the SAG-M bag (reduction of only 93.3 mg/dL [18.8%], if compared to T0), than in the CPDA-1 bag (reduction 129.8 mg/dL [24.1%], compared to T0). This may indicate that, even in smaller concentrations, the glucose consumption in SAG-M bags was kept more constant, being able to maintain ATP levels of red blood cells and extend the storage period. The reduction in glucose levels in the CPDA-1 bags was lower than that described for other species, such as humans (89%) [28], canines (70%) [29], and equines (26%) [12]. According to Harvey and Kaneko [30] the consumption of glucose by erythrocytes in equines is 0.64 mol/h/mL. During storage, the glucose is consumed by anaerobic glycolysis and is the main source of energy for erythrocytes, generating molecules that protect against free radicals, because even during preservation the metabolism of erythrocytes is kept active [31].

The levels of sodium reduced and the concentration of potassium increased over the storage period in both bags. Sodium is an electrolyte present in high concentrations in the extracellular medium, while potassium is found in greater amounts inside the cell [32] Since it is an intracellular ion, the increase in the levels of extracellular potassium represents a reduction in the capacity of the membrane to maintain this ion concentration in the intracellular medium or may even indicate hemolysis [22]. The gradual increase in the K+ concentration in the extracellular medium occurs due to its leakage from the interior of the red cells, and together with a reduction in the sodium this indicates a failure in the functioning of the Na and K pump [24]. In a previous study in horses, the sodium levels found in CPDA-1 bags after 30 days of storage were 158 mmol/L [12], which is higher than the sodium levels (122 mmol/L) found in this study, considering that the storage period was the same. The normal concentration of Na+ in the erythrocytes of a horse is 10.4 mmol/L. The sodium and potassium concentrations should be assessed based on the species, because the intracellular and extracellular levels of these electrolytes vary. In bovines, the concentration of sodium erythrocyte is around 79.1 mmol/L [30], while in donkeys the whole sodium and potassium concentration (Na+/K+) varied between 110 and 114.2 mmol/L, lower than the total value sodium in the erythrocytes of horses (130.4 mmol/L) [33].

As glucose is consumed for ATP production and the consequent maintenance of cell integrity, lactate, the final product of the glycolytic path activity, is accumulated. This provokes acidification of the medium, one of the greatest problems of preservation of erythrocytes in normal conditions [6,23,24].

The relationship between pH and potassium levels in the CPDA-1 and SAG-M bags showed a negative correlation. The sodium and potassium pump is sensitive to changes in temperature, ATP, and pH. The high correlation between pH and potassium concentrations demonstrated that the plasma membrane is directly affected by pH reduction, possibly influencing the potassium migration contained in the intracellular fluid to the extracellular medium [19,34]. 

The reduction in pH is responsible for the commitment of glycolytic enzymes and, consequently, for ATP production and the functioning of the Na+-K+-ATPase. This is an important limiting factor for the viability of erythrocytes. The SAG-M bag was able to maintain a higher concentration of potassium inside the cell, possibly due to the presence of mannitol, which can stabilize the cellular membrane, promoting the impermeability of the erythrocyte membrane while maintaining high intracellular pH levels and reducing hemolysis [6]. The concentration of potassium in the extracellular donkey blood (15.58 mmol/L) was near the values found during the storage of horse blood in CPDA-1 [12], considering the same period of storage. 

Changes occurred in the levels of sodium, potassium, glucose; the number of red blood cells; and pH during the storage of donkey whole blood in CPDA-1 bags and CPD/SAG-M bags, which can possibly have a few implications during small-volume transfusions or transfusions in metabolically stable animals. Nevertheless, it may have serious harmful effects if higher volumes are used in neonates, geriatric patients, and patients with renal or hepatic insufficiency. In these cases, where there are patients at risk the measurement of potassium and pH, for example, in stored blood should be carried out before transfusion, and the sick animals should be systemically monitored during the blood administration [12]. Considering the microbiology results, the presence of bacteria did not influence [12] the pH of the bags, and the blood in both the bags was negative for bacteria growth at all analysis times.

The amount of preservative solution inside the CPD/SAG-M bags is a limiting factor for the evaluation of some variables, since the greater volume of solution in this bag promotes the dilution of some analyzed variables when compared to the CPDA-1 blood bag. Although donkey blood is viable for transfusion after 45 days of storage according to the variables analyzed, further studies are required to evaluate the possible clinical transfusion reaction of these animals when submitted to the allogenic transfusion of fresh or stored whole blood.

## 5. Conclusions

Packed cell volume, plasma hemoglobin, hemolysis level, pH, pCO_2_, pO_2_, glucose, potassium, and microbiological assessment proved to be efficient indicators of blood viability for the donkey species and could be established as parameters of assessment for donkey blood under preservation in CPDA-1 and SAG-M bags. CPDA-1 and SAG-M bags can be used for donkey whole blood conservation, under refrigeration, for up to 42 days.

## Figures and Tables

**Table 1:** Mean and standard deviation values of hematological variables of donkey whole blood stored in CPDA-1 and CPD/SAG-M bags for 42 days.

Variables	Bags	Moments								
		T0	T1	T3	T7	T14	T21	T28	T35	T42
PCV (%)	CPDA-1	32.0 ± 3.3 ^Ab^	31.8 ± 3.1 ^Ab^	32.0 ± 3.3 ^Ab^	32.0 ± 3.3 ^Ab^	32.4 ± 3.1 ^Ab^	32.4 ± 3.1 ^Ab^	32.8 ± 3.3 ^Ab^	33.1 ± 4.5 ^Ab^	35.0 ± 4.4 ^Aa^
	SAG-M	25.1 ± 1.5 ^Bb^	25.0 ± 1.6 ^Bb^	25.0 ± 1.6 ^Bb^	25.2 ± 1.6 ^Bb^	26.0 ± 2.0 ^Bab^	26.1 ± 1.9 ^Bab^	26.7 ± 1.7 ^Bab^	26.5 ± 2.3 ^Bab^	28.2 ± 1.3 ^Ba^
RBC (×10^6^)	CPDA-1	5.9 ± 0.7 ^Aa^	5.3 ± 0.7 ^Aab^	5.2 ± 0.7 ^Aab^	5.3 ± 1.0 ^Aab^	5.0 ± 0.5 ^Aab^	5.1 ± 0.7 ^Aab^	4.9 ± 0.6 ^Aab^	4.8 ± 0.7 ^Ab^	4.8 ± 0.7 ^Ab^
	SAG-M	4.2 ± 0.8 ^Ba^	4.0 ± 0.6 ^Bab^	3.9 ± 0.5 ^Bab^	3.8 ± 0.4 ^Ba^	3.77 ± 0.4 ^Bab^	3.7 ± 0.4 ^Bab^	3.5 ± 0.4 ^Bab^	3.4 ± 0.4 ^Bb^	3.4 ± 0.4 ^Bb^
MCV (fL)	CPDA-1	57.9 ± 6.2 ^c^	59.6 ± 6.3 ^c^	61.2 ± 5.8 ^bc^	60.5 ± 7.3 ^bc^	64.3 ± 3.4 ^abc^	63.7 ± 5.2 ^abc^	66.4 ± 4.7 ^abc^	69.1 ± 5.7 ^ab^	73.1 ± 6.4 ^Ba^
	SAG-M	60.8 ± 8.0 ^d^	62.7 ± 6.2 ^d^	64.1 ± 5.0 ^dc^	66.4 ± 9.8 ^bcd^	69.1 ± 4.3 ^bcd^	70.4 ± 4.2 ^bcd^	75.3 ± 8.5 ^abc^	77.2 ± 10.2 ^ab^	84.1 ± 9.2 ^Aa^
Free Hb (g/dL)	CPDA-1	0.012 ± 0.007 ^c^	0.016 ± 0.008 ^dc^	0.019 ± 0.008 ^bcd^	0.023 ± 0.008 ^bcd^	0.029 ± 0.014 ^abcd^	0.031 ± 0.010 ^abc^	0.035 ± 0.010 ^abc^	0.037 ± 0.009 ^ab^	0.043 ± 0.017 ^a^
	SAG-M	0.007 ± 0.003 ^d^	0.012 ± 0.006 ^cd^	0.014 ± 0.006 ^bcd^	0.029 ± 0.007 ^abcd^	0.024 ± 0.010 ^abcd^	0.025 ± 0.011 ^abc^	0.028 ± 0.014 ^abc^	0.031 ± 0.015 ^ab^	0.036 ± 0.016 ^a^
Haemolysis degree (%)	CPDA-1	0.089 ± 0.07 ^c^	0.111 ± 0,06 ^c^	0.125 ± 0.05 ^bc^	0.149 ± 0.06 ^abc^	0.180 ± 0.09 ^abc^	0.184 ± 0.06 ^abc^	0.206 ± 0.07 ^abc^	0.259 ± 0.10 ^ab^	0.266 ± 0.12 ^a^
	SAG-M	0.061 ± 0.07 ^c^	0.098 ± 0.06 ^bc^	0.125 ± 0.05 ^abc^	0.190 ± 0.06 ^abc^	0.195 ± 0.09 ^abc^	0.207 ± 0.11 ^ab^	0.236 ± 0.11 ^ab^	0.257 ± 0.11 ^a^	0.291 ± 0.11 ^a^
WBC (×10^3^)	CPDA-1	10.8 ± 1.9 ^Aa^	9.4 ± 1.7 ^Aab^	8.8 ± 1.7 ^Aab^	8.2 ± 1.5 ^Aabc^	7.6 ± 1.5 ^Abc^	6.9 ± 1.2 ^Abcd^	5.9 ± 1.3 ^cd^	4.7 ± 1.7 ^d^	4.9 ± 1.3 ^d^
	SAG-M	7.8 ± 0.6 ^Ba^	7.3 ± 0.9 ^Bab^	6.7 ± 0.9 ^Bab^	6.1 ± 1.0 ^Babc^	5.6 ± 1.1 ^Bbcd^	4.7 ± 0.9 ^Bcde^	3.9 ± 0.9 ^de^	3.2 ± 0.7 ^e^	3.5 ± 1.4 ^e^

The different capitalized letters in the columns indicate difference among bags (*p* < 0.05). Different lower-case letters on the line indicate time differences (*p* < 0.05). PCV: packed cell volume; RBC: red blood cell count; MCV: mean corpuscular volume; Hb: hemoglobin; WBC: white blood cell count. CPDA-1: citrate-phosphate-dextrose and adenine; CPD/SAG-M: citrate-phosphate-dextrose, adenine, mannitol, and sodium chloride.

**Table 2:** Mean and standard deviation values of blood gas variables of donkey whole blood stored in CPDA-1 and CPD/SAG-M bags for 42 days.

Variables	Bags	Moments								
		T0	T1	T3	T7	T14	T21	T28	T35	T42
pH	CPDA-1	7.09 ± 0.03 ^a^	7.09 ± 0.05 ^a^	7.08 ± 0.17 ^a^	6.97 ± 0.04 ^ab^	6.93 ± 0.04 ^bc^	6.89 ± 0.04 ^bcd^	6.84 ± 0.05 ^bcd^	6.83 ± 0.05 ^cd^	6.78 ± 0.06 ^d^
	SAG-M	7.10 ± 0.17 ^a^	7.04 ± 0.04 ^ab^	7.01 ± 0.04 ^ab^	6.96 ± 0.04 ^bc^	6.88 ± 0.03 ^cd^	6.84 ± 0.04 ^de^	6.83 ± 0.06 ^def^	6.74 ± 0.03 ^ef^	6.71 ± 0.05 ^f^
pCO_2_ (mmHg)	CPDA-1	65.85 ± 4.9 ^e^	66.38 ± 10.1 ^e^	75.35 ± 9.6 ^Ade^	84.64 ± 6.6 ^Acd^	90.81 ± 8.3 ^bc^	98.69 ± 5.3 ^Aab^	101.4 ± 5.1 ^Aab^	105.9 ± 5.5 ^a^	103.1 ± 10.1 ^ab^
	SAG-M	59.81 ± 6.4 ^d^	58.44 ± 6.5 ^d^	61.70 ± 6.1 ^Bcd^	71.59 ± 5.9 ^Bc^	82.54 ± 3.2 ^b^	87.33 ± 4.9 ^Bb^	85.23 ± 10.1 ^Bb^	101.10 ± 6.7 ^a^	98.51 ± 4.7 ^a^
pO_2_ (mmHg)	CPDA-1	59.25 ± 5.0 ^g^	64.88 ± 10.7 ^fg^	80.00 ± 16.2 ^Afg^	93.88 ± 20.3 ^Aef^	123.00 ± 32.6 ^de^	133.30 ± 28.2 ^Acd^	155.90 ± 20.6 ^Abc^	173.00 ± 16.1 ^ab^	192.60 ± 9.6 ^a^
	SAG-M	56.13 ± 4.3 ^c^	55.88 ± 5.1 ^c^	61.75 ± 20.9 ^Bc^	66.00 ± 31.6 ^Bbc^	82.50 ± 45.9 ^bc^	87.88 ± 38.8 ^Bbc^	122.30 ± 47.6 ^Bab^	150.60 ± 44.6 ^a^	169.40 ± 42.0 ^a^
HCO_3_ (mmol/L)	CPDA-1	20.53 ± 1.6 ^Aab^	20.88 ± 1.9 ^Aa^	20.53 ± 2.3 ^Aab^	20.54 ± 1.5 ^Aab^	20.10 ± 1.7 ^Aab^	19.76 ± 1.8 ^Aab^	18.83 ± 1.6 ^Aabc^	17.71 ± 1.5 ^Abc^	16.13 ± 1.6 ^Ac^
	SAG-M	16.76 ± 1.2 ^Ba^	16.68 ± 1.4 ^Ba^	16.14 ± 1.3 ^Ba^	16.79 ± 1.3 ^Ba^	16.26 ± 1.5 ^Ba^	15.49 ± 1.5 ^Bab^	14.74 ± 1.5 ^Bab^	14.56 ± 1.7 ^Bab^	13.14 ± 1.6 ^Bh^

The different capitalized letters in the columns indicate difference among bags (*p* < 0.05). Different lower-case letters on the line indicate time differences (*p* < 0.05). PO_2_: partial pressure of O_2_; PCO_2_: partial pressure of CO_2_; HCO_3_: bicarbonate. CPDA-1: citrate-phosphate-dextrose and adenine; CPD/SAG-M: citrate-phosphate-dextrose, adenine, mannitol, and sodium chloride.

**Table 3:** Mean and standard deviation values of biochemical variables of donkey whole blood stored in CPDA-1 and CPD/SAG-M bags for 42 days.

Variables	Bags	Moments								
		T0	T1	T3	T7	T14	T21	T28	T35	T42
2,3 DPG (µmol/mL)	CPDA-1	3.48 ± 0.6 ^a^	2.83 ± 2.1 ^ab^	NA	2.61 ± 0.3 ^ab^	2.58 ± 0.6 ^ab^	2.55 ± 0.7 ^ab^	NA	1.71 ± 0.4 ^Aab^	1.63 ± 0.8 ^b^
	SAG-M	3.03 ± 1.2 ^a^	2.16 ± 0.3 ^ab^	NA	2.14 ± 0.5 ^ab^	2.7 ± 0.5 ^a^	2.1 ± 0.7 ^ab^	NA	0.99 ± 0.3 ^Bb^	0.95 ± 0.5 ^b^
Glucose (mg/dL)	CPDA-1	537.9 ± 17.1 ^Aa^	545.0 ± 18.4 ^Aa^	538.6 ± 19.4 ^Aa^	527.9 ± 18.8 ^Aab^	509.9 ± 17.4 ^Aab^	490.0 ± 16.6 ^Abc^	452.4 ± 50.4 ^cd^	419.9 ± 43.0 ^d^	408.1 ± 29.1 ^d^
	SAG-M	494.8 ± 12.3 ^Ba^	496.6 ± 13.1 ^Ba^	487.6 ± 15.5 ^Ba^	480.8 ± 12.2 ^Ba^	472.9 ± 13.6 ^Bab^	454.1 ± 15.2 ^Bbc^	442.6 ± 16.6 ^c^	415.0 ± 20.5 ^d^	401.5 ± 18.0 ^d^
Potassium (mmol/L)	CPDA-1	3.08 ± 0.1 ^Ag^	3.45 ± 0.2 ^Ag^	4.21 ± 0.6 ^Afg^	5.52 ± 1.0 ^Af^	7.86 ± 0.9 ^Ae^	10.63 ± 1.0 ^Ad^	14.13 ± 1.0 ^Ac^	17.58 ± 1.1 ^Ab^	22.64 ± 1.0 ^Aa^
	SAG-M	2.50 ± 0.1 ^Af^	2.77 ± 0.1 ^Af^	3.40 ± 0.3 ^Af^	4.36 ± 0.8 ^Aef^	6.36 ± 1.4 ^Bde^	8.12 ± 1.6 ^Bcd^	9.93 ± 1.9 ^Bbc^	11.70 ± 2.2 ^Bab^	13.64 ± 2.6 ^Ba^
Sodium (mmol/L)	CPDA-1	133.4 ± 1.5 ^Ba^	133.8 ± 1.5 ^Ba^	132.8 ± 1.5 ^Ba^	131.5 ± 1.8 ^a^	129.0 ± 2.6 ^Bab^	126.1 ± 2.8 ^bbc^	122.6 ± 4.3 ^Bc^	122.0 ± 4.1 ^Bc^	121.9 ± 4.0 ^Bc^
	SAG-M	138.8 ± 1.5 ^Aa^	137.8 ± 1.7 ^Aab^	137.9 ± 1.3 ^Aab^	135.1 ± 2.9 ^abc^	134.9 ± 1.7 ^Abc^	133.1 ± 2.4 ^Acd^	130.5 ± 2.7 ^Ad^	129.5 ± 2.5 ^Ad^	129.4 ± 2.7 ^Ad^
LDH (U/L)	CPDA-1	142.6 ± 36.4 ^e^	200.6 ± 50.4 ^Ade^	198.5 ± 47.8 ^Ade^	271.3 ± 61.7 ^Ade^	312.1 ± 93.9 ^Acde^	425.3 ± 140.1 ^Abcd^	573.4 ± 176.6 ^ab^	545.3 ± 249.7 ^abc^	686.0 ± 260.4 ^a^
	SAG-M	110.5 ± 40.0 ^c^	111.1 ± 47.2 ^Bc^	117.9 ± 42.4 ^Bc^	156.4 ± 52.6 ^Bbc^	146.5 ± 20.3 ^Bbc^	160.7 ± 27.7 ^Bbc^	371.8 ± 199.6 ^ab^	458.0 ± 231.2 ^a^	514.0 ± 250.7 ^a^
Lactate (mg/dL)	CPDA-1	25.9 ± 3.5 ^Ad^	26.3 ± 4.4 ^Acd^	30.3 ± 3.7 ^Acd^	45.1 ± 6.5 ^Ab^	58.8 ± 2.8 ^Aa^	67.3 ± 2.9 ^Aa^	36.5 ± 13.7 ^bc^	42.3 ± 2.9 ^b^	40.8 ± 4.0 ^b^
	SAG-M	22.1 ± 2.2 ^Bd^	19.9 ± 1.6 ^Bd^	23.3 ± 2.9 ^Bd^	36.0 ± 4.0 ^Bc^	51.3 ± 4.3 ^Ba^	54.8 ± 3.7 ^Ba^	45.1 ± 1.7 ^b^	43.6 ± 2.3 ^b^	43.0 ± 3.8 ^b^

The different capitalized letters in the columns indicate difference among bags (*p* < 0.05). Different lower-case letters on the line indicate time differences (*p* < 0.05). 2,3 DPG: 2,3 diphosphoglycerate; LDH: lactate dehydrogenase. NA: Not available. CPDA-1: citrate-phosphate-dextrose and adenine; CPD/SAG-M: citrate-phosphate-dextrose, adenine, mannitol, and sodium chloride.

## Data Availability

The raw data is freely available upon request to the corresponding author.
